# Supplementary material for: The Effect of Omega-3 and Omega-6 Polyunsaturated Fatty Acids on the Production of Cyclooxygenase and Lipoxygenase Metabolites by Human Umbilical Vein Endothelial Cells
Source: Nutrients. 2019 Apr 27;11(5):966. doi: 10.3390/nu11050966 (PMC6567278; doi:10.3390/nu11050966)
Supplement: Supplementary file 1 [file nutrients-11-00966-s001.pdf]

Table 1. A 2^4-full factorial design to assess the release of PGE_2, PGE_3, 6-keto-$PGF_{1\alpha}$, Δ17-6-keto-$PGF_{1\alpha}$, LTB_4, RvD_1, 17epi-RvD_1, RvD_2, RvD_3 and RvD_4 into endothelial cell growth basal medium-2 (EBM-2) by HUVEC after exposure to the following PUFA: DHA, EPA, ALA and ARA. The first and last rows (in grey) represent control cell cultures with and without ethanol (-EtOH) that is used to dissolve the PUFA, respectively. The results in picogram units (pg) are separated by a slash and correspond to three independent replicates, except for ALL-ALA that was measured in duplicate.

CODE	DHA	EPA	ALA	ARA	PGE_2	PGE_3	6-keto-$PGF_{1\alpha}$	Δ17-6-keto-$PGF_{1\alpha}$
+EtOH	-1	-1	-1	-1	3.3/4.2/5.1	5.5/8.2/9.2	6.9/7.8/10.5	33.2/36.2/34.2
ALL	+1	+1	+1	+1	90.2/60.5/77.7	56.9/46.6/52.1	65.9/70.7/90.1	189.0/133.4/204.1
ALL-ARA	+1	+1	+1	-1	5.1/5.9/6.2	13.0/13.0/16.9	14.8/12.1/13.1	62.2/59.3/87.7
ALL-ALA	+1	+1	-1	+1	83.0/97.8	59.4/81.5	28.7/38.9	110.0/137.1
DHA+EPA	+1	+1	-1	-1	4.9/6.3/5.5	17.1/12.3/17.4	7.9/9.3/6.5	20.2/18.3/14.0
ALL-EPA	+1	-1	+1	+1	127.4/112.3/119.5	56.1/51.0/75.3	33.2/48.4/46.5	145.3/165.3/151.8
DHA+ALA	+1	-1	+1	-1	3.5/3.0/3.3	6.4/6.8/7.7	19.3/16.1/14.2	58.9/36.5/56.9
DHA+ARA	+1	-1	-1	+1	21.7/29.3/22.3	52.0/73.8/66.1	25.2/24.4/25.7	171.8/135.5/140.8
DHA	+1	-1	-1	-1	5.1/4.1/3.3	9.8/6.9/7.1	14.9/12.4/12.3	22.7/35.8/29.2
ALL-DHA	-1	+1	+1	+1	191.8/174.8/188.0	57.2/61.1/57.2	95.7/81.4/72.7	200.7/195.0/199.2
EPA+ALA	-1	+1	+1	-1	5.5/6.4/8.5	17.8/19.3/15.6	34.4/32.5/31.5	63.7/71.0/96.8
EPA+ARA	-1	+1	-1	+1	409.4/401.5/381.1	119.0/126.1/90.6	53.8/63.9/60.4	532.8/531.6/355.9
EPA	-1	+1	-1	-1	8.5/7.6/7.2	18.0/25.5/19.0	23.2/28.1/19.3	32.1/34.0/45.6
ALA+ARA	**-1**	**-1**	+1	+1	244.2/266.9/221.5	93.2/84.8/57.7	53.1/66.5/53.2	105.3/115.9/117.8
ALA	**-1**	**-1**	+1	-1	7.1/8.0/7.5	6.3/6.6/9.3	33.0/29.1/28.5	33.3/43.3/48.5
ARA	**-1**	**-1**	-1	+1	375.8/380.2/323.1	115.8/147.9/128.6	105.0/115.5/80.5	528.1/699.4/624.3
-EtOH	-1	-1	-1	-1	1.0/1.4/1.0	2.2/3.0/2.5	3.1/4.3/4.0	20.9/18.5/13.5

CODE	LTB_4	RvD_1	17epi-RvD_1	RvD_2	RvD_3	RvD_4
+EtOH	3.5/3.1/4.1	0.0/0.0/0.0	0.0/0.0/0.0	0.0/0.0/0.0	0.6/0.5/0.5	8.7/10.2/12.4
ALL	223.8/182.3/185.6	30.5/31.4/20.9	20.0/15.2/17.6	6.9/6.0/4.5	5.1/4.4/6.8	104.5/77.2/105.9
ALL-ARA	97.7/98.6/116.2	14.7/12.0/18.3	15.0/23.9/21.3	1.7/1.9/1.1	1.4/1.9/1.6	10.5/11.5/13.2
ALL-ALA	122.2/111.0	15.2/14.5	26.6/23.0	3.5/3.3	4.6/4.5	49.2/47.9
DHA+EPA	36.9/54.5/42.8	21.0/16.0/20.0	2.1/2.5/1.8	14.6/10.6/11.4	6.5/6.8/7.0	31.7/36.0/40.9
ALL-EPA	170.2/190.3/197.5	12.8/17.4/18.6	30.0/33.1/35.0	7.7/6.4/6.2	2.7/2.8/2.5	70.0/75.1/66.5
DHA+ALA	38.7/39.3/32.7	18.0/15.0/17.1	13.7/17.2/11.4	1.9/2.1/1.7	9.8/8.6/6.4	61.4/55.1/52.6
DHA+ARA	63.1/71.6/61.1	29.7/31.6/38.1	40.5/33.7/30.0	7.7/5.3/6.4	6.4/4.1/6.1	54.5/64.7/65.8
DHA	15.8/10.7/16.5	19.7/16.0/13.5	10.8/10.0/14.5	2.2/1.8/1.6	7.4/5.1/4.8	45.3/41.1/49.4
ALL-DHA	55.6/60.2/60.7	2.1/3.2/3.2	0.0/0.0/0.0	0.8/0.7/0.6	1.4/1.6/1.9	0.0/0.0/0.0
EPA+ALA	27.9/24.0/36.0	0.0/0.0/0.0	0.3/0.2/0.3	1.0/0.9/0.8	1.9/1.7/1.6	1.6/1.1/1.2
EPA+ARA	81.6/72.9/70.7	1.5/1.8/1.3	0.5/0.6/0.6	0.8/0.5/0.8	1.6/2.0/1.4	3.6/3.9/4.1
EPA	23.5/24.8/20.0	3.0/4.7/3.3	4.8/4.0/4.5	0.8/0.7/0.7	0.7/0.6/0.6	1.5/1.8/1.4
ALA+ARA	55.7/53.5/51.3	1.1/1.6/1.2	0.0/0.0/0.0	0.6/0.6/0.8	0.7/0.8/0.8	2.6/2.1/2.3
ALA	15.4/11.9/19.1	2.2/2.4/2.0	2.2/2.8/2.5	1.0/1.2/1.5	0.6/0.7/0.8	1.0/1.1/1.4
ARA	27.1/34.7/27.9	0.0/0.0/0.0	1.8/2.5/1.6	1.0/1.2/1.2	2.1/2.0/3.0	3.0/3.3/3.8
-EtOH	2.6/1.8/2.0	0.1/0.1/0.1	0.0/0.0/0.0	0.0/0.0/0.0	0.5/0.5/0.5	12.8/11.9/14.5

The term CODE is used to summarized the various conditions dictated by the design. For example, ALL-DHA is the a condition where EPA, ALA and ARA are at level +1 and DHA is at level -1.

Table S2 Significant effects computed by using the data in Table S1.

	p-values									
	Prostaglandins		Prostacyclins		Leukotriene	Resolvins				
Main effects	PGE_2	PGE_3	6-keto-$PGF_{1\alpha}$	Δ17-6-keto-$PGF_{1\alpha}$	LTB_4	RvD_1	17epi-RvD_1	RvD_2	RvD_3	RvD_4
DHA	0.000	0.000	0.000	0.000	0.000	0.000	0.000	0.000	0.000	0.000
EPA	0.793	0.895	0.050	0.839	0.000	0.794	0.000	0.002	0.158	0.000
ALA	0.000	0.000	0.000	0.000	0.000	0.254	0.208	0.000	0.008	0.021
ARA	0.000	0.000	0.000	0.000	0.000	0.000	0.000	0.005	0.427	0.000
Interactions										
DHA×EPA	0.115	0.223	0.087	0.789	0.001	0.075	0.000	0.002	0.006	0.013
DHA×ALA	0.000	0.001	0.003	0.000	0.000	0.130	0.007	0.000	0.022	0.000
DHA×ARA	0.000	0.000	0.000	0.000	0.000	0.000	0.000	0.013	0.000	0.000
EPA×ALA	0.000	0.384	0.000	0.000	0.022	0.002	0.338	0.000	0.438	0.444
EPA×ARA	0.830	0.001	0.333	0.216	0.385	0.286	0.000	0.000	0.001	0.000
ALA×ARA	0.000	0.000	0.969	0.000	0.000	0.530	0.000	0.000	0.211	0.000
DHA×EPA×ALA	0.796	0.614	0.118	0.040	0.044	0.000	0.006	0.000	0.001	0.781
DHA×EPA×ARA	0.132	0.056	0.000	0.329	0.013	0.111	0.002	0.000	0.000	0.000
DHA×ALA×ARA	0.000	0.001	0.000	0.000	0.000	0.442	0.000	0.000	0.053	0.000
EPA×ALA×ARA	0.000	0.562	0.000	0.019	0.000	0.000	0.023	0.000	0.000	0.000
DHA×EPA×ALA ×ARA	0.523	0.460	0.003	0.044	0.042	0.000	0.000	0.000	0.000	0.000

DHA: docosahexaenoic acid; EPA: eicosapentaenoic acid; ALA: α-linolenic acid; ARA: arachidonic acid

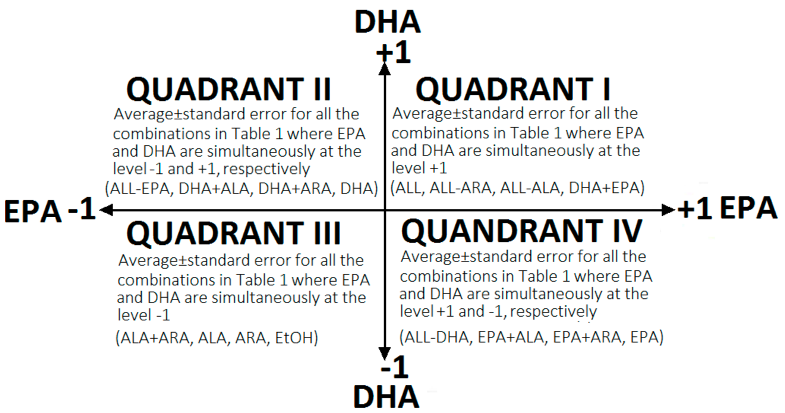

CODE	PUFA ω-3			ω-6		
	DHA	EPA	ALA	ARA		
ALL	+1	+1	+1	+1	Quadrant-I	(EPA, DHA)= (+1,+1)
ALL-ARA	+1	+1	+1	-1	Quadrant-I	
ALL-ALA	+1	+1	-1	+1	Quadrant-I	
DHA+EPA	+1	+1	-1	-1	Quadrant-I	
ALL-EPA	+1	-1	+1	+1		
DHA+ALA	+1	-1	+1	-1		
DHA+ARA	+1	-1	-1	+1		
DHA	+1	-1	-1	-1		
ALL-DHA	-1	+1	+1	+1	Quadrant-IV	(EPA, DHA)= (+1,-1)
EPA+ALA	-1	+1	+1	-1	Quadrant-IV	
EPA+ARA	-1	+1	-1	+1	Quadrant-IV	
EPA	-1	+1	-1	-1	Quadrant-IV	
ALA+ARA	-1	-1	+1	+1		
ALA	-1	-1	+1	-1		
ARA	-1	-1	-1	+1		
EtOH	-1	-1	-1	-1		

Figure 1. How to construct the two-factor interaction diagrams in Tables 3-4. The pair of PUFA are allocated on perpendicular axes by forming four quadrants. The different combinations of +1 and -1 values in each quadrant are assigned according to the 2^4 factorial design. For example, when studying the interaction EPA and DHA they are designated as x-axis and y-axis respectively. The quadrant I (levels +1,+1) contains the average value of all the experiments containing simultaneously both EPA and DHA. According to the factorial design, the conditions that fulfil this requirement are those designated by the code: ALL, ALL-ARA, ALL-ALA and DHA+EPA. In a similar manner, the quadrant IV (levels +1, -1) contains the average value of all the experiments containing EPA but not DHA. According to the factorial design, the conditions that fulfil this requirement are those designated by the code: ALL-DHA, EPA+ALA, EPA+ARA and EPA.
